# Transcranial Direct Current Stimulation and Mindfulness for Cognitive and Mood Recovery in Stroke Survivors: A Pilot Randomized Controlled Study

**DOI:** 10.1155/srat/3893469

**Published:** 2025-05-24

**Authors:** Atekeh Mosannaei Najibi, Sama Rahnemayan, Alireza Poursoleimani, Rasoul Heshmati, Mohammad Ali Nazari, Erfan Golshan Shali, Ehsan Nasiri, Mehdi Farhoudi

**Affiliations:** ^1^Faculty of Psychology, Marand Azad University, Marand, Iran; ^2^Neurosciences Research Center (NSRC), Tabriz University of Medical Sciences, Tabriz, Iran; ^3^Department of Psychology, University of Tabriz, Tabriz, Iran; ^4^Department of Neuroscience, Faculty of Advanced Technologies in Medicine, Iran University of Medical Sciences, Tehran, Iran

**Keywords:** ACE, BDI, cognitive rehabilitation, depression, mindfulness, poststroke depression, stroke, tDCS

## Abstract

**Background:** Cognitive impairments and depression are common after stroke. Noninvasive treatments like transcranial direct current stimulation (tDCS) and mindfulness-based interventions have shown potential for improving these outcomes, though their effects on stroke survivors remain unclear. This study is aimed at evaluating the efficacy of mindfulness and tDCS in enhancing cognitive function and alleviating depression in stroke survivors.

**Methods:** This randomized controlled trial, conducted from July 2021 to July 2022, included 30 stroke survivors divided into three groups: mindfulness (*n* = 5), tDCS (*n* = 14), and control (*n* = 11). Cognitive function was measured using Addenbrooke's Cognitive Examination-III (ACE-III), and depression was assessed using the Beck Depression Inventory-II (BDI-II) before and after interventions. The tDCS group received 10 sessions of anodal stimulation, and the mindfulness group underwent eight weekly sessions of mindfulness-based stress reduction. Data were analyzed using paired *t*-tests for within-group comparisons and ANOVA for between-group differences.

**Results:** The tDCS group showed significant improvement in cognitive function, with ACE-III scores increasing by 9.14 ± 8.24 points (*p* = 0.02). Fluency and orientation scores also improved significantly in this group (*p* < 0.001 and *p* = 0.01, respectively). No significant cognitive changes were observed in the mindfulness group. Depression scores (BDI-II) did not change significantly in any group.

**Conclusions:** tDCS significantly improved cognitive performance, particularly in fluency and orientation, while mindfulness showed no significant cognitive or depression-related effects. Future studies should explore the long-term impact of these interventions in stroke rehabilitation.

**Trial Registration:** ClinicalTrials.gov identifier: IRCT20090716002195N3

## 1. Introduction

Stroke, a major neurological disorder caused by cerebrovascular complications, is a leading cause of disability and death worldwide. It ranks as the fourth leading cause of death among women and the fifth among men [1, 2]. Globally, stroke-related mortality rates range from 250 to 400 per 100,000 individuals [3]. Beyond mortality, the more profound impact of stroke lies in the long-term disabilities it causes, including motor deficits, cognitive decline, mood disorders, and an increased risk of complications such as rehospitalization and falls. These sequelae substantially reduce the quality of life and life expectancy of stroke survivors [4].

Among the poststroke complications, cognitive impairments and mood disorders such as poststroke depression (PSD) are particularly prevalent and debilitating. Cognitive deficits may affect memory, attention, executive functions, and concentration [5], while PSD—affecting over 30% of stroke patients in the first year—significantly worsens overall functional outcomes and increases mortality risk [6–9]. Given the profound impact of these complications on recovery, effective interventions targeting both cognitive deficits and mood disorders are essential for improving stroke rehabilitation outcomes.

Traditional treatments for PSD, such as pharmacotherapy and psychotherapy, often have limited efficacy and come with potential side effects [10, 11]. As a result, there is a growing interest in alternative therapeutic approaches that can enhance both cognitive function and mood without significant adverse effects [12, 13]. Two promising interventions are mindfulness-based interventions and transcranial direct current stimulation (tDCS) [14, 15]. While these therapies have shown potential benefits for mood and cognitive function in other populations, their specific effects in stroke survivors remain underexplored [16].

Mindfulness, the practice of maintaining conscious awareness of the present moment, has demonstrated efficacy in enhancing memory, attention, executive function, and processing speed [17]. Furthermore, mindfulness-based therapies, including meditation and yoga, have been reported to contribute positively to stroke rehabilitation [18]. Mindfulness-based interventions operate through mechanisms of focused attention, emotional regulation, and increased awareness of present-moment experiences, which have been shown to enhance functional connectivity in brain regions involved in attention, memory, and emotional control, such as the prefrontal cortex and anterior cingulate cortex [19, 20]. These neural changes are thought to contribute to improvements in both cognitive functioning and mood regulation.

tDCS, in contrast, modulates cortical excitability by delivering a low-intensity electrical current to specific areas of the brain. Anodal stimulation typically increases neuronal excitability, whereas cathodal stimulation decreases it. When applied over the prefrontal cortex, tDCS can enhance neuroplasticity and facilitate synaptic efficiency in networks related to executive function, attention, and memory [21, 22]. This neuromodulatory effect may support recovery in cognitive and emotional domains in various clinical populations [23]. Several clinical trials have indicated that anodal tDCS over the prefrontal cortex may enhance cognitive recovery in stroke patients [24].

Despite these promising findings, there is limited research directly comparing the effects of mindfulness and tDCS on cognitive and emotional rehabilitation in stroke survivors. Therefore, this study is aimed at evaluating the efficacy of these two interventions in addressing both cognitive impairments and depression in stroke patients. By exploring these noninvasive and accessible treatment options, this research seeks to provide insights into potential rehabilitation strategies that could improve the quality of life for stroke survivors.

## 2. Methods

This randomized controlled clinical trial was conducted from July 2021 to July 2022 at Imam Reza Hospital, Tabriz, on stroke patients who met the inclusion and exclusion criteria. The study was conducted during the second wave of the COVID-19 pandemic. To ensure participant and staff safety, all sessions were conducted in compliance with local and institutional COVID-19 guidelines. These included mask-wearing, hand hygiene protocols, physical distancing, and regular disinfection of equipment and surfaces. Additionally, participants and staff were screened for COVID-19 symptoms prior to each session, and vaccination status was recorded when available.

The study is aimed at assessing the efficacy of mindfulness-based interventions and tDCS on cognitive enhancement and depression alleviation among stroke survivors. Participants were randomly assigned to one of three groups: mindfulness, tDCS, or control. Randomization was performed using a computer-generated random sequence, prepared in advance by an independent statistician not involved in participant recruitment or assessment. Allocation concealment was ensured using sequentially numbered, sealed, opaque envelopes; these envelopes were opened only after the participant's enrollment to determine assignment to the mindfulness, tDCS, or control group.

Blinding of the participants and experts was not possible due to the nature of the interventions. Inclusion criteria for the study included unilateral brain lesions, a minimum of 3 months since stroke onset, age between 30 and 75, ability to attend follow-up visits, and literacy. Exclusion criteria included refusal to provide informed consent, speech-related left hemisphere lesions, a history of multiple strokes, or pre-existing psychiatric illness.

A formal sample size calculation was not performed prior to the study due to its pilot nature and limited eligible patient pool during the recruitment period. The sample size was based on the number of participants who met the inclusion criteria and consented to participate during the study period. The findings from this trial will inform sample size calculations for future, larger-scale studies.

### 2.1. Interventions

All study participants continued to receive routine standard medical care for stroke as prescribed by their treating physicians, which could include antihypertensives, antiplatelet agents, statins, and other medications in accordance with clinical guidelines. However, no participant received pharmacological treatment specifically for depression or cognitive impairment during the study period.

Participants in the mindfulness group received mindfulness training based on Kabat–Zinn's Mindfulness-Based Stress Reduction (MBSR) program. This program consisted of eight weekly sessions, each lasting 2.5 h. The sessions were led by certified experts with a minimum of 10 years of experience in mindfulness practice.

tDCS was administered using the Oasis Pro device (Mind Alive Inc., Canada). For the tDCS group, anodal tDCS was applied to the left lateral prefrontal cortex (F3), with cathodal stimulation on the right lateral forehead (F4). The stimulation was administered at 2 mA for 20-min sessions applied 5 days/week on weekdays over a 2-week period, for a total of 10 sessions. Participants in the control group did not receive any additional interventions during the study period.

### 2.2. Cognitive and Depression Measures

Cognitive function was assessed using the Addenbrooke's Cognitive Examination-III (ACE-III), which evaluates attention and orientation, memory, fluency, language, and visuospatial abilities. This assessment was conducted both before and after the intervention phase to measure any changes in cognitive performance [25]. Depression levels were measured using the Beck Depression Inventory-II (BDI-II), a widely used instrument to gauge the severity of depression, with scores ranging from mild to severe [26]. Both cognitive and depression assessments were performed pre- and postintervention to track the impact of the interventions on participants' cognitive and emotional states.

### 2.3. Statistical Analysis

SPSS version 25 was used for data analysis. Continuous variables, such as BDI-II and ACE-III scores, are presented as mean ± standard deviation (SD). For within-group comparisons of pre- and postintervention BDI-II and ACE-III scores, paired *t*-tests were employed. Changes in specific subdomains (orientation, memory, fluency, language, and visuospatial abilities) were also analyzed for each group.

Due to the relatively small sample sizes, we tested the data for normality using the Shapiro–Wilk/Kolmogorov–Smirnov test. Where data met the assumptions of normal distribution and homogeneity of variances, parametric tests were used. To compare the effects of interventions between the control, mindfulness, and tDCS groups, one-way analysis of variance (ANOVA) was conducted. Post hoc analyses followed where significant differences were found to determine between-group variations. Statistical significance was set at *p* < 0.05.

To provide clinically relevant effect estimates, we calculated the number needed to treat (NNT) and number needed to harm (NNH) for each intervention compared to the control group. A “clinically significant improvement” was defined as a ≥ 5-point increase in ACE-III total score after intervention.

As individual participant response rates were not available, the proportion of participants estimated to achieve this threshold in each group was calculated using the observed group means and SDs, assuming a normal distribution. The absolute risk reduction (ARR) was defined as the difference in these proportions between groups. NNT was calculated as the reciprocal of ARR (NNT = 1/ARR). NNH was similarly calculated for any clinically significant worsening or adverse outcomes; as no such events were recorded in any group, NNH was not applicable in this sample.

## 3. Results

In this randomized clinical trial, 54 patients enrolled in the study after checking for eligibility criteria among 89 candidates and were randomly assigned to three groups: tDCS intervention (*n* = 18), mindfulness-based intervention (mindfullness; *n* = 18), and control (*n* = 18). All participants in the tDCS and control groups received their allocated intervention, while four participants in the mindfulness group did not start the intervention. During the course of the interventions, attrition occurred across all groups. In the tDCS group, four participants discontinued the intervention. In the mindfulness group, five participants discontinued during the intervention after initially starting, resulting in a final sample of five completing the study. In the control group, seven participants discontinued the intervention phase. In total, data from 14 participants in the tDCS group, 5 in the mindfulness group, and 11 in the control group were included in the final analysis.

**Figure 1:**
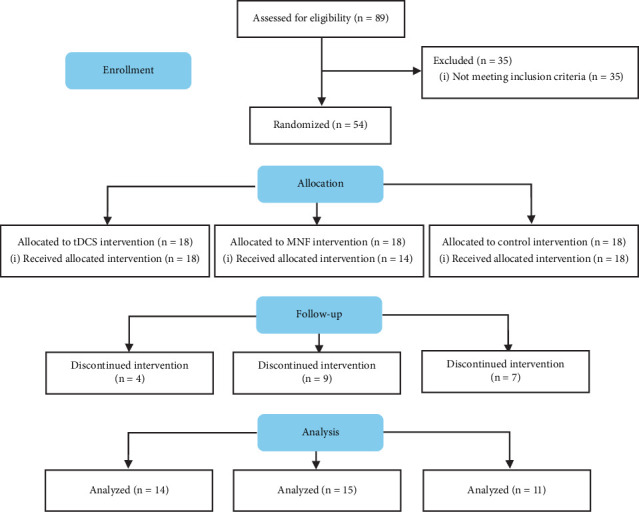
Participants' enrollment process.

In the mindfulness group, 80% were male, and 20% were female. Similarly, males dominated the tDCS group with 85.7%, while the control group had 63.6% males and 36.4% females.  Figure 1 shows the flow diagram of the study enrollment.

No serious adverse events were reported during the course of the tDCS intervention. Mild and transient side effects, such as mild scalp tingling and slight redness at the electrode site, were reported by two participants but resolved spontaneously without further intervention. No participants withdrew from the study due to tDCS-related adverse effects.

### 3.1. Depression and Cognitive Function Before and After Interventions

Table 1 shows the pre- and postintervention comparison of BDI-II and ACE-III scores for each group.

None of the groups exhibited significant changes in depression symptoms after the interventions. The control group's BDI-II scores slightly increased (*p* = 0.66), the tDCS group showed a mild reduction (*p* = 0.76), and the mindfulness group remained unchanged (*p* = 1.00).

**Figure 2:**
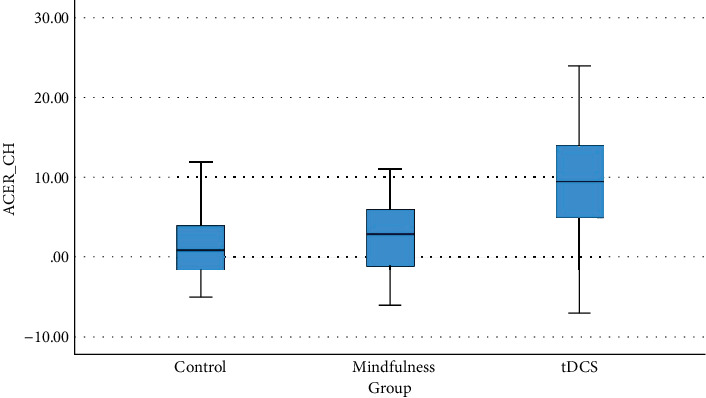
Distribution of ACE-III change in each group.

Cognitive improvements were evident in the tDCS group, with a significant increase in ACE-III scores from 63.57 ± 15.07 to 72.71 ± 11.35 (*p* = 0.001). The mindfulness group experienced a nonsignificant improvement, from 74.40 ± 19.29 to 77.00 ± 16.21 (*p* = 0.42), while the control group saw no substantial change (*p* = 0.33).  Figure 2 illustrates the distribution of ACE-III score changes across the three groups.

### 3.2. Subdomain Comparisons

Each intervention's effect on specific cognitive subdomains was also assessed (Table 1). Significant improvements were observed in the tDCS group (*p* = 0.01), while the mindfulness and control groups did not show notable changes (*p* = 0.52 and *p* = 0.10, respectively). Although tDCS showed a tendency to improve memory scores, this result was marginal (*p* = 0.06). The mindfulness group also showed nonsignificant memory gains (*p* = 0.06), while the control group experienced a slight reduction (*p* = 0.31).

**Figure 3:**
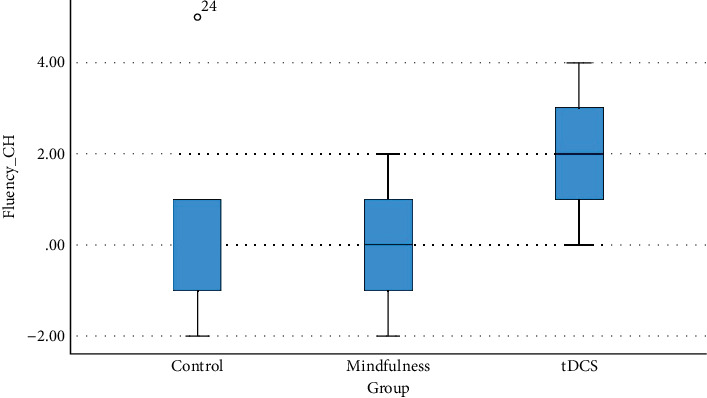
Distribution of fluency change in each group.

The tDCS group had a significant improvement in fluency (*p* < 0.001), unlike the mindfulness and control groups, which showed no significant changes (*p* = 1.00 and *p* = 0.55, respectively).  Figure 3 shows the distribution of fluency score changes in each group. Finally, while tDCS improved language scores (*p* = 0.01), there were no notable changes in visuospatial skills across all groups.

### 3.3. Between-Group Comparisons

According to Table 2, the tDCS group demonstrated more substantial cognitive improvements than the mindfulness and control groups. ACE-III score changes were significantly higher in the tDCS group (9.14 ± 8.24) compared to the control and mindfulness groups (*p* = 0.02). The tDCS group also showed a notable improvement in fluency compared to the other groups (*p* = 0.04). Other domains, such as memory and visuospatial abilities, did not show significant between-group differences.

Based on ACE-III change scores, we estimated that 69% of tDCS group participants, 23% of control, and 36% of mindfulness group achieved clinically significant cognitive improvement (defined as ≥ 5-point increase in ACE-III). The calculated ARR for tDCS vs. control was 0.46, corresponding to a NNT of 3. No patients in any group experienced clinically significant worsening or adverse events; therefore, the NNH could not be determined in this sample.

### 3.4. Gender Influence

Both male and female participants showed similar responses to the interventions, except for BDI-II and orientation changes in the tDCS group, which differed significantly between male and female participants (*p* = 0.01 and *p* = 0.006, respectively).

## 4. Discussion

This randomized controlled trial is aimed at investigating the effects of mindfulness-based interventions and tDCS on cognitive enhancement and depression alleviation among stroke survivors. The findings provide valuable insights into the potential role of these interventions in poststroke rehabilitation, particularly in addressing cognitive impairments. While the effects on depression were minimal across all groups, tDCS demonstrated a significant impact on cognitive function, especially in the domains of orientation and fluency.

The most notable finding of this study was the significant improvement in ACE-III scores among participants in the tDCS group. The tDCS intervention led to a marked increase in overall cognitive performance, particularly in orientation and fluency subdomains. This aligns with previous research suggesting that tDCS, when applied to the prefrontal cortex, enhances cortical excitability and facilitates neuroplasticity, which is critical for cognitive recovery following stroke [27, 28]. The improvement in fluency is particularly noteworthy, as this cognitive domain is often challenging to recover poststroke due to the involvement of complex linguistic and executive processes.

In this study, the NNT for tDCS versus the control group was 3, suggesting high clinical utility; however, estimates are based on a cutoff derived from distributional assumptions due to a lack of individual-level response data. The significant between-group differences in ACE-III score changes, as demonstrated by the ANOVA, further support the efficacy of tDCS in cognitive rehabilitation. Compared to the control and mindfulness groups, participants in the tDCS group experienced greater cognitive gains, particularly in orientation, memory, and fluency. These results are consistent with the growing body of literature suggesting that tDCS can promote cognitive recovery by modulating brain activity in regions associated with attention, memory, and executive function [24, 29].

In contrast to tDCS, the mindfulness intervention did not yield significant improvements in cognitive outcomes, as indicated by the nonsignificant changes in ACE-III scores and subdomains. Although mindfulness-based interventions have been shown to enhance cognitive function in other populations [30], the absence of significant improvements in this study may be due to several factors. One possibility is that the duration and intensity of the mindfulness intervention, consisting of eight sessions, may not have been sufficient to induce measurable changes in cognitive performance among stroke survivors. Additionally, the mindfulness group was the smallest (*n* = 5), which may have limited the statistical power to detect significant changes.

Another factor could be the specific cognitive domains affected by mindfulness. While mindfulness has been shown to improve attention and executive function [31, 32], these domains may not have been as severely impaired in the participants or as responsive to the intervention as those targeted by tDCS. Further research with larger sample sizes and longer intervention periods is needed to fully explore the potential cognitive benefits of mindfulness in stroke rehabilitation.

Interestingly, neither tDCS nor mindfulness had a significant effect on depression symptoms, as measured by BDI-II. This finding contrasts with previous studies suggesting that both tDCS and mindfulness can alleviate depressive symptoms [33, 34]. Several factors may explain this discrepancy. First, the baseline BDI-II scores of participants in the mindfulness group were relatively low (10.40 ± 8.01), which may have created a floor effect, limiting the potential for further reductions in depression scores. Second, the severity of depression may not have been sufficiently high in the study population to observe substantial changes following the interventions.

Moreover, the relatively short duration of the interventions, particularly mindfulness, may not have been sufficient to produce meaningful changes in mood. While mindfulness-based interventions have shown promise in improving mood and emotional regulation in other populations, longer-term interventions may be necessary to achieve similar effects in stroke survivors.

The analysis of gender differences within the tDCS group revealed no significant variation in response to the intervention, either in cognitive outcomes or depression scores. Both male and female participants exhibited similar patterns of improvement, suggesting that gender did not modulate the effectiveness of tDCS. This finding is inconsistent with previous research indicating that the benefits of tDCS are influenced by gender [35, 36].

### 4.1. Study Limitations

This study has several limitations that should be considered. First, the relatively small sample size, particularly in the mindfulness group and also female participants, may have limited the ability to detect significant differences in cognitive and depression outcomes. However, in order to alleviate the effect of inequality among female and male participants, we looked at the gender differences, which revealed no differences among the two genders. Additionally, the small sample size in each group may affect the appropriateness of parametric statistical tests due to potential violations of normality assumptions. Although normality was assessed, the power of such tests and the generalizability of the findings are constrained. Future studies should aim for larger sample sizes to enable more robust statistical analyses. Second, the lack of blinding in the study design, while difficult to achieve in this context, may have introduced bias in the assessment of outcomes. Third, the study duration may have been too short to fully capture the long-term effects of mindfulness and tDCS on cognitive and emotional recovery.

## 5. Conclusion

In conclusion, this study provides preliminary evidence that tDCS can significantly enhance cognitive function, particularly in orientation and fluency, among stroke survivors. Mindfulness, while beneficial in other populations, did not produce significant cognitive or mood improvements in this trial, likely due to its shorter intervention period and smaller sample size. Neither intervention significantly reduced depression symptoms, indicating that further research is needed to explore the optimal duration and combination of therapies for addressing both cognitive and emotional impairments poststroke.

## Figures and Tables

**Table 1:** Comparison of BDI-II and ACE-III scores and subdomain before and after intervention in each group.

**Score**	**Group**	**Before intervention**	**After intervention**	**p** ** value**
BDI-II	Control	22.81 ± 11.08	23.90 ± 11.59	0.66
	tDCS	18.71 ± 11.85	17.64 ± 14.26	0.76
	Mindfulnes	10.40 ± 8.01	10.40 ± 10.16	1.00

ACE-III	Control	63.54 ± 13.96	65.00 ± 15.93	0.33
	tDCS	63.57 ± 15.07	72.71 ± 11.35	0.001
	Mindfulnes	74.40 ± 19.29	77.00 ± 16.21	0.42

Orientation	Control	14.00 ± 3.00	15.36 ± 2.33	0.10
	tDCS	13.92 ± 3.38	16.35 ± 1.08	0.01
	Mindfulnes	16.00 ± 2.44	16.60 ± 0.89	0.52

Memory	Control	16.27 ± 4.38	15.00 ± 6.84	0.31
	tDCS	13.71 ± 6.20	16.50 ± 4.48	0.06
	Mindfulnes	16.00 ± 2.54	18.80 ± 2.28	0.06

Fluency	Control	5.36 ± 2.76	5.72 ± 2.61	0.55
	tDCS	4.07 ± 2.81	5.92 ± 3.04	<0.001
	Mindfulnes	9.00 ± 3.39	9.00 ± 3.80	1.00

Language	Control	19.27 ± 5.17	20.45 ± 5.12	0.20
	tDCS	19.14 ± 3.59	21.21 ± 3.78	0.01
	Mindfulnes	20.40 ± 7.63	21.00 ± 4.84	0.69

Visuospatial	Control	8.63 ± 4.03	8.45 ± 3.47	0.81
	tDCS	12.71 ± 3.17	12.71 ± 3.09	1.00
	Mindfulnes	13.00 ± 4.00	11.60 ± 6.18	0.43

**Table 2:** Comparison of the BDI-II and ACE-III scores and subdomain changes in all groups.

**Criteria**	**Control ** **m** **e** **a** **n** ± **SD**	**Mindfulness ** **m** **e** **a** **n** ± **SD**	**tDCS ** **m** **e** **a** **n** ± **SD**	**p** ** value**
BDI-II score changes	1.09 ± 8.14	0.00 ± 4.74	1.07 ± 13.18−	0.87
ACE-III score changes	1.45 ± 4.80	2.60 ± 6.50	9.14 ± 8.24	0.02
Attention changes	1.36 ± 2.50	0.60 ± 1.94	2.42 ± 3.10	0.39
Memory changes	1.27 ± 4.00−	2.80 ± 2.48	2.78 ± 5.26	0.07
Language changes	1.18 ± 2.89	0.60 ± 3.13	2.07 ± 2.73	0.55
Fluency changes	0.36 ± 1.96	0.00 ± 1.58	1.85 ± 1.40	0.04
Visuospatial changes	0.18 ± 2.52−	1.40 ± 3.64−	0.00 ± 2.38	0.59

## Data Availability

The data that support the findings of this study are available from the corresponding author upon reasonable request.
